# Chronic stress does not impair liver regeneration in rats

**DOI:** 10.1186/s40340-015-0011-8

**Published:** 2015-12-02

**Authors:** Kasper J. Andersen, Anders R. Knudsen, Ove Wiborg, Frank V. Mortensen

**Affiliations:** Department of Surgical Gastroenterology L, Aarhus University Hospital, 8000 Aarhus C, Denmark; Institute of Clinical Medicine – Translational Neuropsychiatry Unit, Aarhus University Hospital, Aarhus, Denmark

**Keywords:** Liver regeneration, Surgery, Chronic stress, Depression, Rats

## Abstract

**Background:**

Although wound healing is a simple regenerative process that is critical after surgery, it has been shown to be impaired under psychological stress. The liver has a unique capacity to regenerate through highly complex mechanisms. The aim of this study was to investigate the effects of chronic stress, which may induce a depression-like state, on the complex process of liver regeneration in rats.

**Methods:**

Twenty rats were included in this study. The animals received either a standard housing protocol or were subjected to a Chronic Mild Stress (CMS) stress paradigm. All rats underwent a 70 % partial hepatectomy (PHx). The animals were evaluated on postoperative day 2 or 4. Blood samples were collected to examine circulating markers of inflammation and liver cell damage. Additionally, liver tissues were sampled to evaluate liver weight and regeneration rate.

**Results:**

None of the animals died during the study. There were no differences between in body weight, liver weight, liver regeneration rate or biochemical markers at any time during the study.

**Conclusion:**

The results of this study indicate that stress and the induction of depression-like state do not affect the process of liver regeneration after 70 % hepatectomy in rats.

## Background

Wound healing is a simple regenerative process that is critical after surgery. Poor wound healing increases the risk of infections and hernias and can lengthen the hospital stay for many patients. The wound healing process progresses through several stages. The first stage is the inflammatory stage. This stage involves vasoconstriction, blood coagulation, and the release of chemoattractant factors and cytokines [1]. The second step is wound remodeling, which may continue for weeks or months [2]. Previous studies have shown that psychological stress has a negative impact on wound healing in both animals and humans [3].

The liver has a unique regenerative capacity after hepatic resection. Wound healing and liver regeneration are both complex healing processes involving many different pathways. Liver regeneration results in proliferation and hypertrophy of the residual liver lobes while the regenerating liver simultaneously maintains homeostasis for the body [4]. Surgical liver resection is increasingly being performed for both primary and secondary cancers worldwide. Due to their recent malignant diagnosis and the procedures described above, these patients are often under psychological stress.

The aim of the present study was to investigate the effects of stress and depression on the complex wound healing process of liver regeneration in a rat model.

## Methods

### Animals and ethics

This entire animal study was performed after approval of the Danish Animal Experiment Inspectorate, Copenhagen, Denmark under the license 2012-15-2934-00591. The experiments were conducted in accordance with the *Guide for the Care and Use of Laboratory Animals* published by the National Institute of Health, USA. Male Wistar rats were obtained from Taconic (Borup, Denmark) and were acclimatized to the animal facility for one week prior to initiation of the protocol. The non-stressed control animals were housed in standard animal laboratories with a temperature maintained at 23 °C and an artificial 12-h light–dark cycle. The animals had free access to food (Altromin) and water. The rats subjected to the stress paradigm were exposed to unpredictable mild stressors as described in the experimental design section. All rats were monitored daily for changes in weight, behavior, and physical appearance.

### Experimental design

**Fig. 1:**
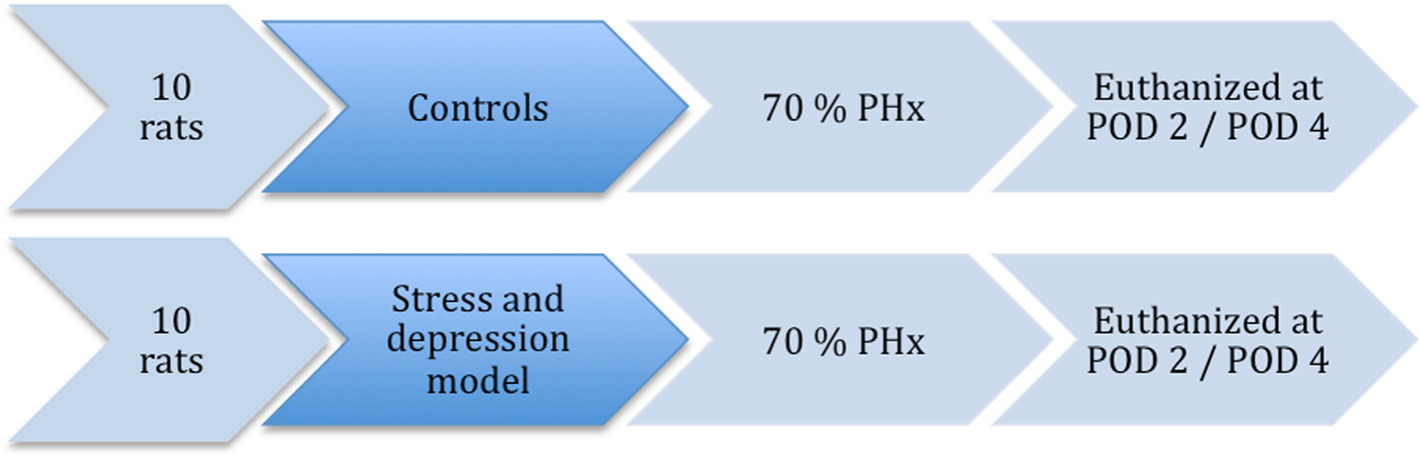
Flowchart. The flowchart of the experiment

In this study, twenty male Wistar rats, ten weeks old, were randomized to either the non-stressed control group or the stress exposed group. After chronic stress exposure, or a corresponding waiting time for the control group, the animals underwent a 70 % partial hepatectomy. The animals were evaluated on postoperative day (POD) 2 or 4 (Fig. 1). These days were chosen based on a previously conducted study, which showed day 2 and 4 to be key points in rat liver regeneration [5].

### Treatment

**Fig. 2:**
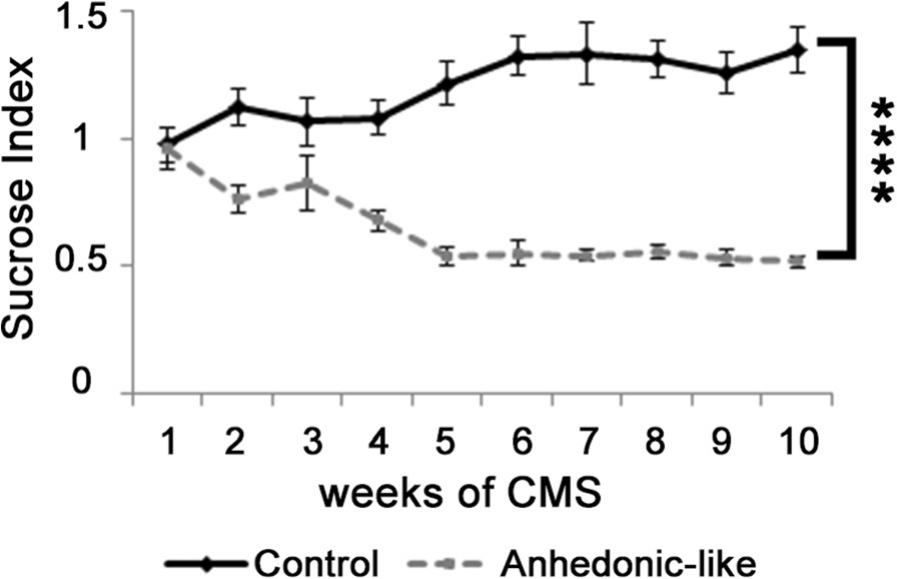
Sucrose Index. Sucrose consumption data indexed to individual baseline values. Data are given as mean +/− SEM. Control rats (*n* = 10) and anhedonia-like rats (*n* = 10). Anhedonia-like rats have significantly lower sucrose intake than control rats shown by RM-ANOVA (F(1,18) = 67.105, *p* < 0.0001). Pair-wise differences were demonstrated at weeks 2, 4–10 by Bonferroni’s post hoc analysis (*p* < 0.0001)

The rats were subjected to the CMS paradigm for 11 weeks. The applied micro stressors were; intermittent illumination, stroboscopic light, grouping, food or water deprivation, damped bedding and cage tilting. Stressors were applied consequentially each lasting 10–14 h. Voluntary intake of a 1.5 % sucrose solution is used as a weekly readout on hedonic capacity or depression-like status. After 2–3 weeks of stress exposure the depression-like rats have a significantly lower sucrose intake (*p* < 0.0001) as illustrated in Fig. 2. The protocol is described in details elsewhere [6]. Rats were approximately 5 months old at the time of resection. The animals had a bodyweight of approximately 400 g.

### Anesthetics and analgesia

All surgical procedures were performed using inhalation anesthesia. The anesthesia induction was performed in a glass cylinder filled with a mixture of oxygen (2.0 l/min), N_2_O (0.5 l/min) and 4 % isoflurane (Forene, Abbott Laboratories, UK). During surgery the anesthesia was maintained with 2 % isoflurane, oxygen, and N_2_O administered through a nasal mask.

### Surgical procedure

The animals were placed in a supine position on a heated pad and then a transverse abdominal incision was made and the liver was mobilized. A partial liver resection was performed using a previously described technique [7]. Briefly, the bases of the median and left lateral lobes were ligated before the lobes were resected resulting in resection of 70 % of the liver. The abdomen was closed with 4–0 absorbent sutures in two layers using single knots. Before surgery, the animals received a subcutaneous injection of the long lasting NSAID Carprofen (Rimadyl, Pfizer Animal Health, Exton, USA) at a dose of 5 mg/kg in 1.0 ml isotonic saline.

### Evaluation

The rats were randomized for euthanisation on either POD 2 or POD 4. Animals were re-anaesthetized, weighed and a laparotomy was performed through the previous incision. Blood samples were collected from the heart by cannulation. All rats were subsequently euthanized by cervical dislocation. The regenerated liver was then mobilized and removed. The liver weight was recorded. The animal body weight and any morbidity and mortality were recorded daily throughout the study period.

### Biochemical analysis

Blood was collected from the heart at sacrifice. The blood was then processed and stored at −80 °C until analysis. The alanine aminotransferase (ALAT), alkaline phosphatase (ALP), albumin (ALB), gamma-glutamyl transferase (GGT) and bilirubin (BR) levels were measured using the Modular P system (Roche Diagnostics, Mannheim, Germany).

The rat acute phase protein α-2-macroglobulin was evaluated using a specific ELISA kit (Immunology Consultants Laboratory, Newberg, Oregon, USA) according to the manufacturer’s instructions. All samples were assayed in duplicate. The assays exhibited intra- and inter-assay coefficients of variance below and 10 %, respectively.

### Liver weight and regeneration rate

The liver weight changes were evaluated using the hepatic regeneration rate (RR). The RR is defined as the liver weight per 100 g of the body weight at euthanasia/preoperative estimated liver weight per 100 g of the body weight x 100 using the following formula:

$$ RR = \frac{\left(\frac{LWm}{100gBW}\right)sac}{\left(\frac{LWc}{100gBW}\right)pre}\cdot 100 $$

LWm is the measured liver weight at euthanasia and LW_C_ is the preoperative calculated liver weight. The preoperatively estimated total liver weight was calculated from the resected liver weight. After removing 70 % liver tissue the LWc was estimated as 100 percent: LW_C_ = (Weight of 70 % rec / 70) x 100.

### Statistical analysis

All statistical analyses were performed in Prism 6 for Mac OS X (1994 – 2014) GraphPad Software, Inc. The data are presented as means and p-values < 0.05 considered significant. The Mann–Whitney test was used to compare groups. For statistical analysis of sucrose data we applied Repeated Measures ANOVA followed by Bonferroni’s Multiple Comparison Test for post hoc analysis.

## Results

### Mortality

There were no animal deaths during the study.

### Body weight

**Fig. 3:**
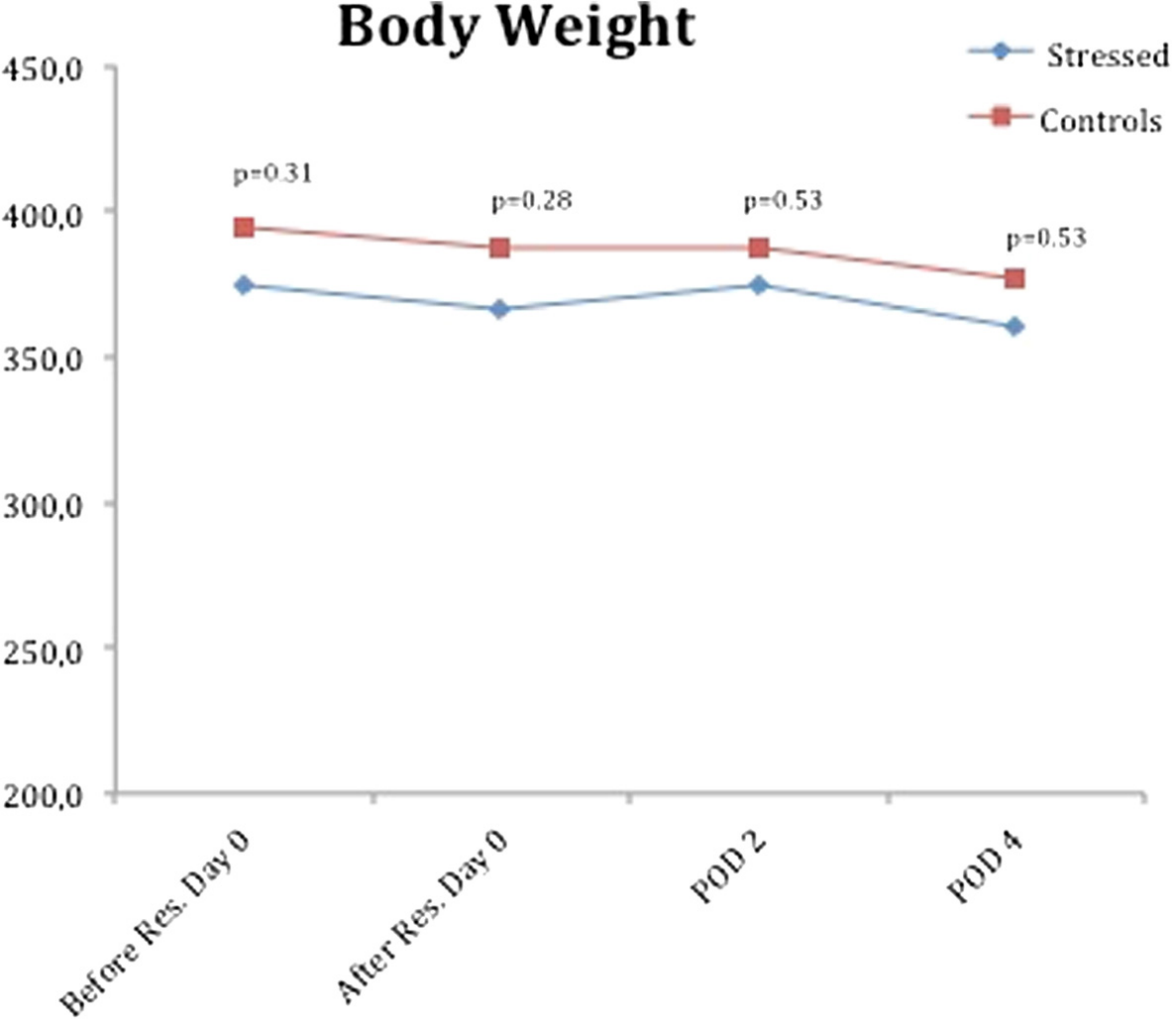
Body weight. Mean body weight for each group of animals. The blue line marks the treatment group and the red line marks the control animals

The animal bodyweights showed lower weight in the CMS group. However, this difference was not significant. Rats in the CMS group had a mean body weight of 374 g (256–426 g) after the CMS-model and before resection. The rats in the control group had a mean body weight of 394 g (337–437 g) before resection. There was no weight loss observed during the regenerative period in any group (Fig. 3).

### Liver weight

**Fig. 4:**
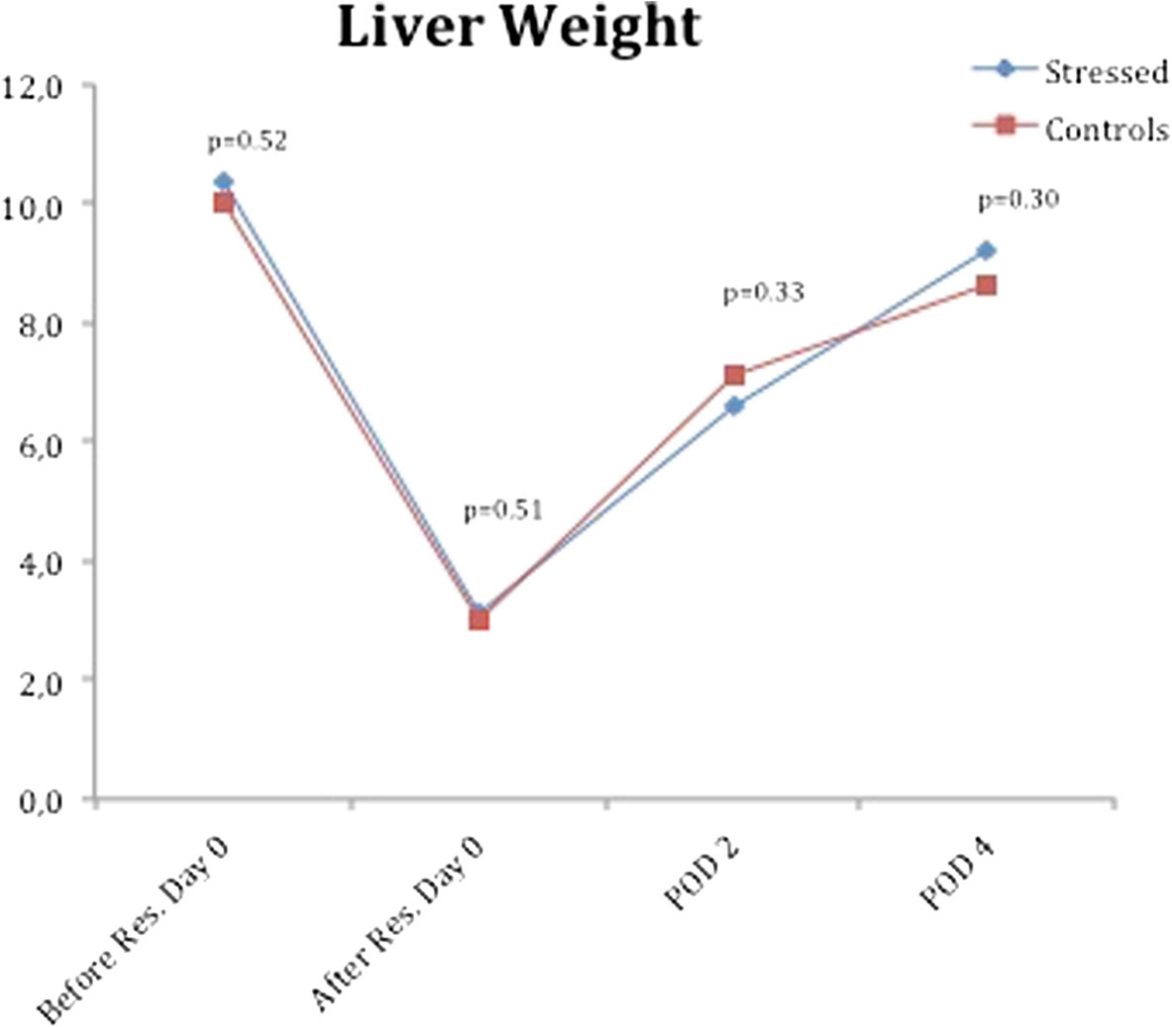
Liver weight. Mean liver weight for each group of animals. The blue line marks the treatment group and the red line marks the control animals

The mean weight of the resected 70 % liver in the CMS rats was 7.3 g (6.6–8.7), which led to an estimated median total liver weight (100 %) of 10.4 g (9.4–12.4). In the control rats, the mean weight of the resected 70 % liver was 7.0 g (5.5–8.1) and led to an estimated median total liver weight (100 %) of 10.0 g (7.9–11.6). The gains in liver weight during the regenerative period for each group are shown in Fig. 3. There was major growth noted during the postoperative period for both groups. The liver weights in both groups approached the baseline values on POD 4. There was no significant difference between the two groups at any time (Fig. 4).

### Liver regeneration rate

**Fig. 5:**
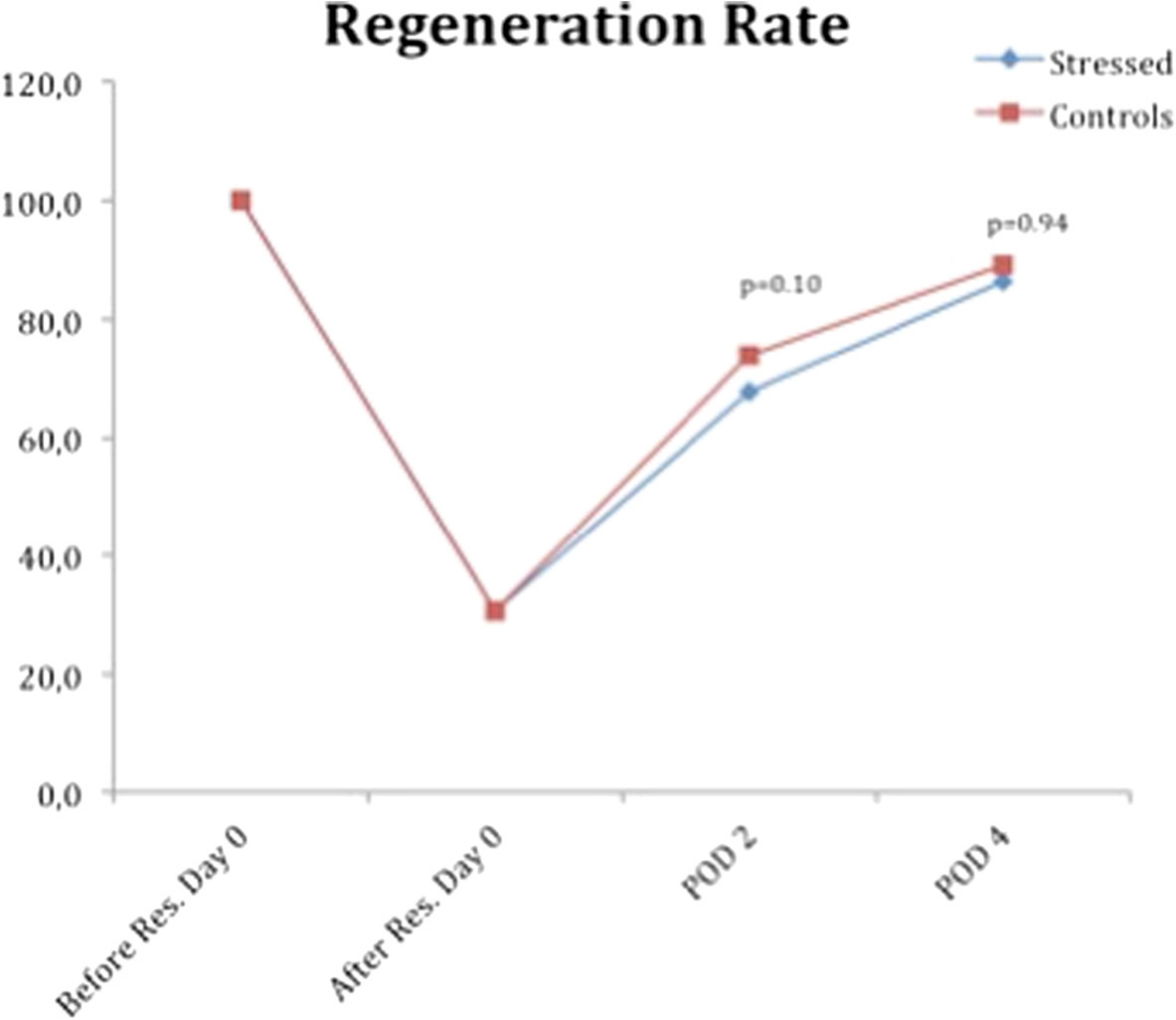
Regeneration Rate. Mean regeneration rate for each group of animals. The blue line marks the treatment group and the red line marks the control animals

The RR showed similar patterns to those observed in the liver weight curves, and regrowth occurred during the study period and approximated the baseline values at POD 4. For the control rats, the RR of 89 was reached at POD 4. At this time, the RR for the CMS animals was 86. There was no significant difference found between groups at any time (Fig. 5).

### Biochemistry

**Fig. 6:**
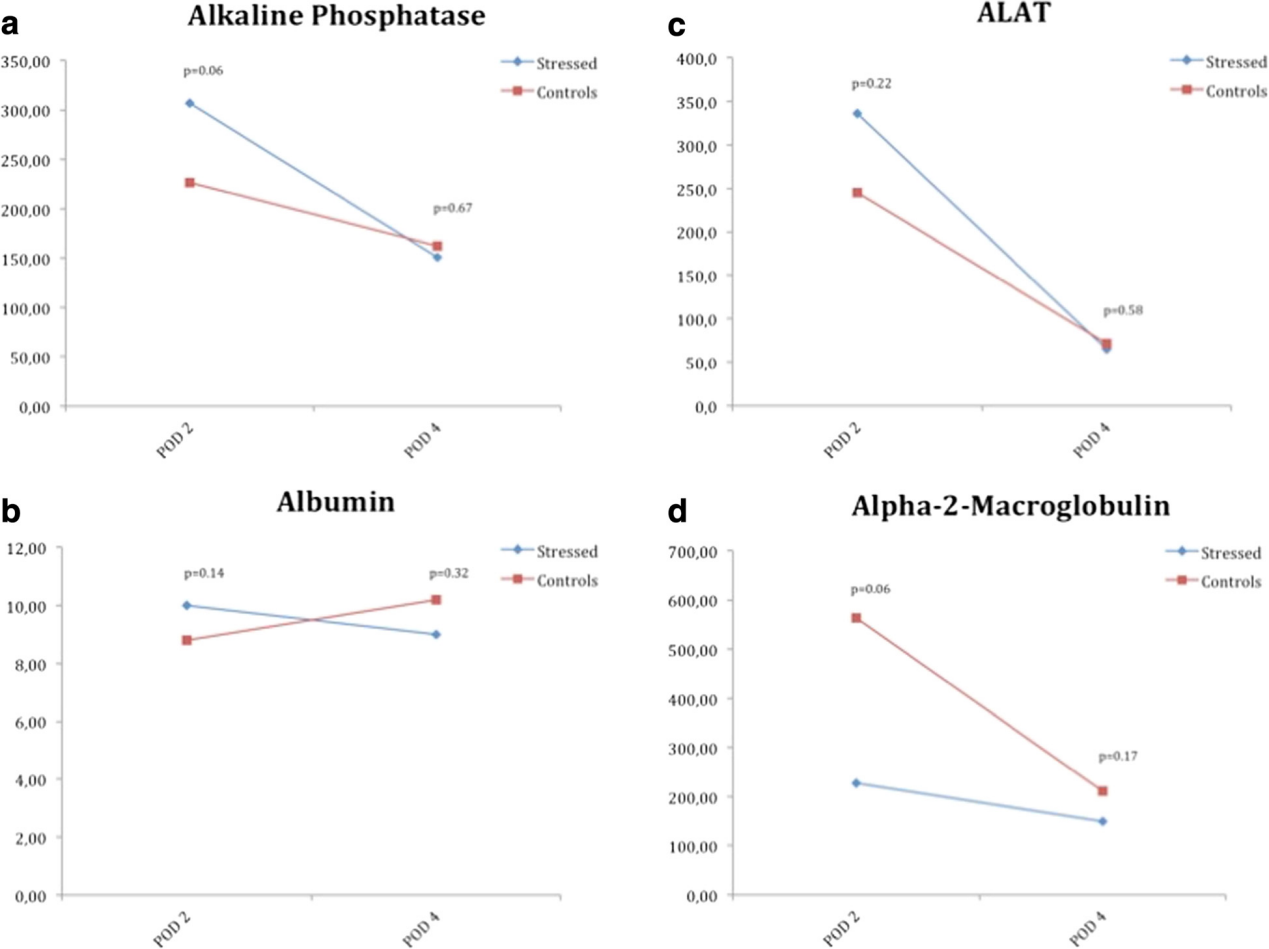
Biochemical markers. Mean alkaline phosphatase (**a**), bilirubin (**b**), alanine aminotransferase (**c**), and alpha-2-macroglobulin (**d**) for each group of animals. The blue line marks the treatment group and the red line marks the control animals

There was no difference in ALP, ALB, ALAT or Alpha-2-Macroglobulin levels at any time between groups (Fig. 6a-d). BR and GGT were under the detection limit at all times.

## Discussion

In this study, we investigated the effect of stress and depression on liver regeneration in rats. We found that depression induced by the CMS paradigm did not affect the complex wound healing process of liver regeneration after partial hepatectomy.

This study is the first to investigate the impact of stress and associated depression on a complex process such as liver regeneration. There was no impact from stress and depression on liver regeneration based on liver growth by absolute liver weight and regeneration rate. Our results are in contrast to several studies on the process of simple wound healing. It has previously been shown that stress and depression have a negative effect on simple wound healing [8, 9]. In the study by Padgett et al., the authors demonstrate mice subjected to restraint stress healed a standardized 3.5 mm full-thickness punch biopsy wound on average 27 % slower than control mice not exposed to stress [8]. Bosch et al. replicated the impact of negative emotions on mucosal wound healing in humans. Among 193 healthy undergraduate students who received a 3.5 mm wound on the hard palate, individuals reporting high levels of depressive symptoms were 3.6 times more likely to be classified as slow healers than less dysphoric students [9]. In another study by Padgett et al., the authors showed that psychological stress activates the hypothalamic-pituitary-adrenal and the sympathetic-adrenal-medullary axes [10]. The enhanced production of glucocorticoids and catecholamines can directly influence several components of the healing process. Furthermore, substantial evidence from both animal and human studies indicates that physiological stress responses can retard the initial inflammatory phase of wound healing [11]. In addition to directly modulating physiological responses to skin damage in humans, stress can also indirectly influence wound repair by promoting the adoption of health-damaging behaviors. Individuals who experience greater levels of stress are more likely to increase their alcohol and tobacco use, decrease their participation in physical activity, experience sleep disturbances, and make poorer diet choices than individuals reporting less distress [12, 13]. These negative health behavior practices can then augment the detrimental impact of stress on physiological healing processes [14].

Liver regeneration is a highly sophisticated process involving some of the same mechanisms as wound healing. However, there are several important differences. In a typical wound healing scenario the injury to the tissue results in disruption of capillary vascular networks and extravasation of blood, which is then accompanied by the local release of coagulation factors, platelets, growth factors, etc. [15]. This is clearly not the case following 2/3 PHx. We surgically removed three liver lobes with minimal damage to the residual two lobes and no extravasation of blood occurred. Although there was only minimal damage to the residual liver tissue, there were substantial changes in hepatic blood flow patterns. Several studies in the literature have suggested that the early hemodynamic changes after PHx are important. Although there was no extravasation of whole blood, the hemodynamic alterations after PHx induced a global spectrum of events across the entire remnant liver [4]. The tissues involved in wound healing have well-described phases that occur in a consistent manner. Thus, there is no need to account for whole body homeostasis. Conversely, liver regeneration is a very complex and well-orchestrated phenomenon that requires the participation of all mature liver cell types. The process is associated with signaling cascades involving growth factors, cytokines, matrix remodeling, and several feedback systems that stimulate and inhibit growth related signals. Provided that the upper limit of app max 75 % liver resection is respected, the liver can restore any lost mass and adjust its size to the organism while simultaneously providing full support for body homeostasis during the entire regenerative process. In situations when hepatocytes or biliary cells are blocked from regeneration these cell types can function as facultative stem cells for each other. This capacity permits complete liver regeneration. Wound healing has been demonstrated compromised in CMS animals. In the present study we could not demonstrate a negative impact of chronic mild stress on liver regeneration. This could be explained by evolutionary mechanisms ensuring ‘protection’ of this crucial capability.

Difference in bodyweight is a reliable marker of acute stress in animals and is crucial in studying animals after liver resections [16]. In this study, both groups showed identical progression of bodyweight. These data indicate the CMS and non-stressed control animals responded similarly to the acute physiological stress of surgery.

The liver enzymes ALAT, ALP, BR and alpha-2-M have traditionally been used as markers of hepatic injury [17–19]. We found there were no differences in liver parameters between groups.

Acute phase proteins have been shown to be elevated in both humans and animals subjected to psychological stress [20, 21]. Our data however, did not show any significant differences between the CMS group and the control group after liver resection. There was however, a tendency towards alpha-2-M being higher in the control group although the dynamics was identical. The observed levels for alpha-2-macroglobulin could be a consequence of the surgical stress put onto the animals, however the acute phase response seem in a way to be paralyzed in stressed animals as We did not collect blood samples before surgery. Thus, there might have been a difference in blood alpha-2-macroglobulin prior to surgery.

Cytokines are known to be early markers of liver regeneration, but are also known to fluctuate over time [22]. We did not measure these parameters in the present setup, as it was only possible to draw one blood sample from each rat at the time of sacrifice. We did not want to expose the animals to additionally stress as this could interfere with the main variable of the present study, i.e. liver regeneration.

We used the Chronic Mild Stress model [6, 23–26] to induce a depression-like condition in the animals. The chronic mild stress (CMS) model is one of several animal model of stress-induced depression. It aims to model a chronic depressive-like state that develops gradually over time in response to stress. CMS involves exposing animals to a series of mild and unpredictable stressors (periods of food and water deprivation, changes in illumination, changes of cage mates, and other similar individually innocuous manipulations) for at least 3–4 weeks. The model has been reported to cause long lasting changes of behavioral, neurochemical, neuroimmune and neuroendocrine variables as well as structural brain changes most importantly affecting reward functions. These functions include increased intracranial self-stimulation thresholds and decreased voluntary intake of sweet solutions reflecting anhedonia. These alterations are reversed by chronic, but not acute antidepressant treatment [6, 23–26]. The advantages of this model are the predictive validity (behavioral changes are reversed by chronic treatment with a wide variety of antidepressants), face validity (several important symptoms of depression have been reproduced), and construct validity (CMS causes a generalized decrease in responsiveness to rewards). However, there is a common practical difficulty in performing CMS experiments because they are labor intensive, require additional space, and take a long time.

It is well known that many patients with a cancer diagnosis develop stress and depression [27, 28]. The results of the present study suggest that patients who undergo liver resections can experience chronic stress without a negative impact on liver regeneration. However, one should be cautious when translating results from animal studies. Therefore, we need further studies to examine this issue in humans.

## Conclusion

In conclusion, the present study showed that stress-induced depression does not affect the complex process of liver regeneration after 70 % hepatectomy in rats.
